# Impact of Ultrasonographic Features Indicative of Malignancy on Tumor Advancement in Thyroid Cancer—A Single-Center Study

**DOI:** 10.3390/cancers17172822

**Published:** 2025-08-28

**Authors:** Michał Miciak, Krzysztof Jurkiewicz, Natalia Kalka, Maja Reiner, Szymon Biernat, Dorota Diakowska, Beata Wojtczak, Krzysztof Kaliszewski

**Affiliations:** 1 Department of General Surgery, University Centre of General and Oncological Surgery, Faculty of Medicine, Wroclaw Medical University, 50-556 Wrocław, Poland; krzysztof.jurkiewicz@student.umw.edu.pl (K.J.); natalia.kalka@student.umw.edu.pl (N.K.); maja.reiner@student.umw.edu.pl (M.R.); szymon.biernat@student.umw.edu.pl (S.B.); 2 Doctoral School, Wroclaw Medical University, 50-345 Wrocław, Poland; 3 Division of Medical Biology, Faculty of Nursing and Midwifery, Wroclaw Medical University, 50-368 Wrocław, Poland; dorota.diakowska@umw.edu.pl; 4 Department of Endocrine Surgery, University Centre of General and Oncological Surgery, Faculty of Medicine, Wroclaw Medical University, 50-556 Wrocław, Poland; beata.wojtczak@umw.edu.pl

**Keywords:** thyroid diagnostics, ultrasound imaging, malignancy features, hypoechogenicity, microcalcifications, cancer management

## Abstract

Background: Ultrasonography is frequently used preoperatively to assess thyroid nodules. Hypoechogenicity, microcalcifications, high vascularity, or irregular tumor shape suggest malignancy. Methods: This is a retrospective analysis of 724 patients from 2008 to 2024 who underwent surgery for TC. Preoperative data, ultrasonographic findings, and histopathological results were collected. Ultrasonographic features indicative of possible malignancy included hypoechogenicity, microcalcifications, high vascularity, and irregular tumor shape. These were correlated with histopathologically seen extrathyroidal extension, capsular and vascular invasion, and lymph node metastasis. Results: A statistically significant association was seen for each of the evaluated ultrasonographic features (*p* < 0.05). More advanced TC had a greater number of suspicious ultrasonographic features averaging 3.05 to 3.12. Microcalcifications, high vascularity, and irregular tumor shape showed a strong correlation (r > 0.7) with all histopathological features. Hypoechogenicity had a strong correlation with lymph node metastasis and a moderate correlation (r = 0.5–0.7) with other features. Conclusions: Ultrasonographic features predict the likelihood of histopathological extrathyroidal extension, capsular and vascular invasion, and lymph node metastasis.

## Simple Summary

This study of 724 patients between 2008 and 2024 with confirmed thyroid cancer (TC), comparing their ultrasound findings with surgical histopathology.

## 1. Introduction

Ultrasonography is a common imaging modality in thyroid cancer (TC). Nodule characteristics (hypoechogenicity, microcalcifications, high vascularity, irregular margins, size > 1 cm) and lymphadenopathy suggest malignancy [1]. Ultrasonographic findings are classified using Thyroid Imaging Reporting and Data System (TIRADS), most commonly in ACR-TIRADS and EU-TIRADS versions. Shear wave elastography is also sometimes used, with high sensitivity and specificity [2,3]. Cytology, via a fine-needle aspiration biopsy (FNAB), is evaluated using the Bethesda System for Reporting Thyroid Cytopathology (2023) [4,5].

This study of TC reports the correlation of ultrasonographic features with histopathological features.

## 2. Materials and Methods

This retrospective study included 724 patients from 2008 to 2024 who underwent surgery for TC. Preoperative data, ultrasonographic findings, and histopathological results were collected. Ultrasonographic features indicative of possible malignancy included hypoechogenicity, microcalcifications, high vascularity, and irregular tumor shape. Histopathological features indicative of TC advancement included extrathyroidal extension, capsular and vascular invasion, and lymph node metastasis (N+). The impact of ultrasonographic features on the presence of aggressive tumor characteristics was analyzed.

### 2.1. Statistical Analysis

Qualitative data were expressed as counts and percentages, while quantitative data were presented as medians with interquartile ranges, reflecting the non-normal distribution. The Shapiro–Wilk test was employed to evaluate the normality of quantitative variables. Group differences were examined using the Mann–Whitney U test for continuous variables, and the chi-square test was applied to compare categorical variables. Relationships between variables were analyzed using Spearman’s rank correlation, appropriate for non-parametric data. All statistical analyses were conducted using Statistica version 10.0 (StatSoft Inc., Tulsa, OK, USA). A *p*-value < 0.05 amounted to a statistically significant difference between the variables.

### 2.2. Study Group Overview

The study group is shown in Table 1. A total of 97% were Bethesda V–VI, and 195 required a second surgery. All patients had thyroid ultrasound and FNAB under ultrasound guidance. Some pN and pM are not known as routine prophylactic lymphadenectomy was not performed.

## 3. Results

### 3.1. Distribution of Ultrasonographic and Histopathological Features

**Figure 1:**
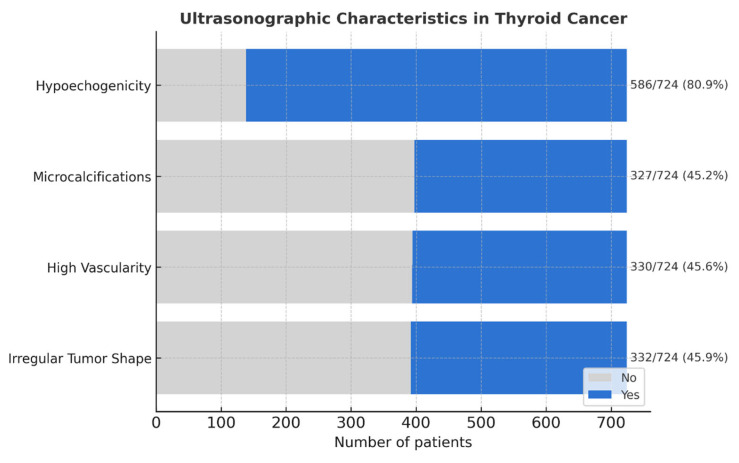

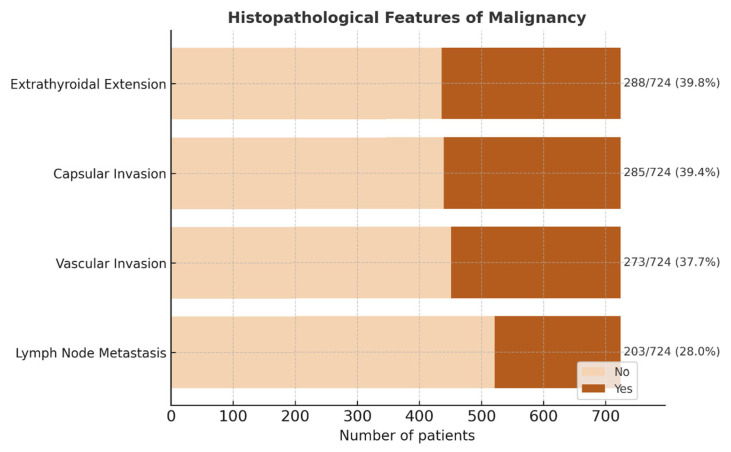
Overview of ultrasonographic and histopathological TC features across the study group.

The ultrasonographic features indicative of malignancy in TC include hypoechogenicity, microcalcifications, high vascularity in the center of the nodule, and irregular tumor shape (or margins). The majority of records (81%) had hypoechogenic lesions. Microcalcifications, high vascularity, and irregular shapes were observed in ~45%. The histopathological features we evaluated included extrathyroidal extension, capsular invasion, vascular invasion (each ~38%), and N+ (28%). The data are summarized in Figure 1.

### 3.2. Association Between Ultrasonographic and Histopathological Features

There was a statistically significant (*p*-value < 0.05 for all) relationship between the suspicious ultrasound features and the more concerning histopathological features. The results are presented in Table 2.

### 3.3. Impact of the Average Number of Ultrasonographic Features

An analysis of the number of suspicious ultrasound features showed that patients with malignant characteristics presented, on average, a higher number of studied ultrasonographic features. The average number of suspicious ultrasonography features in patients with concerning histopathological features was 3.05–3.12, compared with 2.70–2.88 of those without. The data are presented in Table 3.

### 3.4. Cross-Correlation Analysis

**Figure 2:**
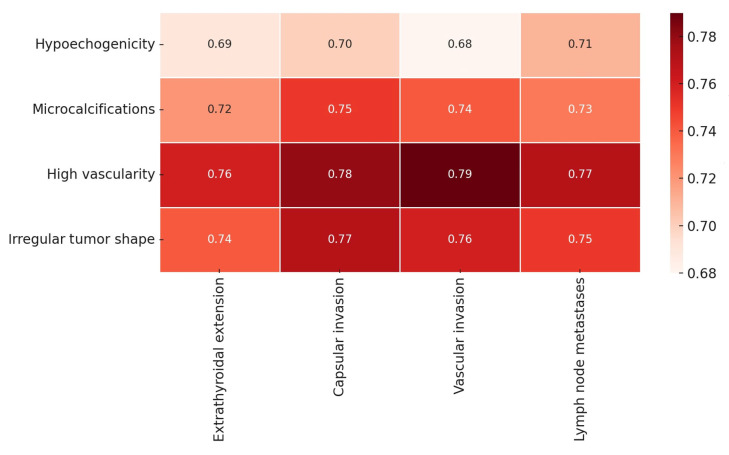
Correlations between ultrasound and histopathology determined using Spearman’s correlation test (r). Note: correlations between variables: 0–0.5, weak; 0.5–0.7, moderate; >0.7, strong.

There is a strong correlation of suspicious ultrasonographic features with concerning histology (Figure 2). For hypoechogenicity, a strong correlation was seen with N+, and a moderate correlation was seen with extrathyroidal extension, capsular invasion, and vascular invasion. For microcalcifications, high vascularity, and irregular tumor shape, strong correlations were observed with all the histopathological features. An increase in the number of suspicious ultrasonographic features significantly elevates the likelihood of malignant characteristics.

## 4. Discussion

This study confirms that ultrasonographic features of thyroid nodules (microcalcifications, high vascularity, irregular lesion shape, and nodule hypoechogenicity) correlate with histopathological features (such as extrathyroidal extension, vascular invasion, thyroid capsular invasion, and lymph node metastases).

Hypoechogenic nodules were present in ~81% of TCs, like other series (71–99%) [6,7,8,9,10]. We observed moderate positive correlations between hypoechogenicity and extrathyroidal extension, capsular invasion, and vascular invasion (r = 0.68–0.7), similar to reports of 42% capsular invasion and 78% vascular invasion in hypoechoic nodules [11,12]. A strong correlation between hypoechogenicity and N+ central compartment (r = 0.71) has also been highlighted in the literature [13,14]. Microcalcifications were observed in 45% of TC cases, consistent with literature reports ranging from 34% to 59% [15,16,17]. This feature is highly specific but moderately sensitive. In some rare cases, TCs have been reported without a distinct nodule, with microcalcifications being the sole abnormality on ultrasound [18,19].

We observed strong correlations between microcalcifications and all histopathological features analyzed (r = 0.72–0.75). Literature reports vary, linking microcalcifications to selected histopathological features, perineural invasion, elevated vascular endothelial growth factor (VEGF) levels, or increased risk when present in more than five foci [20,21,22]. For lymph node metastases, rates of microcalcifications are 27–36%, usually in combination with other unfavorable ultrasonographic features [23,24,25,26].

Ultrasonographic TC features suggestive of malignancy should not be evaluated in isolation [13,14,23,24,25,26,27,28]. Our results are in line with the hypothesis that no single ultrasound feature reliably distinguishes benign from malignant thyroid lesions. Many studies note coexistent features [6,8,9,10,11,12,13,14,15,20,21,22,23,26,27,28,29,30,31,32,33,34,35,36,37,38,39,40]. Patterns of high vascularity, particularly increased central nodule vascularity, were observed in 46% of cases in our study. The literature reports 17% to 92%, with a meta-analysis reporting ~50%, with the variability deriving, in part, from different criteria [29,30,41]. We also observed a similar proportion for irregular tumor shapes (46%), the same as in a literature review (47%). This parameter has been described as highly linked with TC, with specificity ranging from 83% to 97% [31,32,33,42]. In our study, both high vascularity and irregular tumor shape showed strong correlations with aggressive histological features of TC, including extrathyroidal extension (r = 0.74–0.76), capsular and vascular invasion (r = 0.76–0.79), and lymph node metastases (r = 0.75–0.77). Similar associations have been reported elsewhere, with vascularity being a key marker [27,28,34,35,43,44,45]. We saw the strongest correlation between vascularity and vascular invasion. Reported rates of lymph node metastases reach 70%, with an overall malignancy risk of up to 86% in thyroid nodules presenting high vascularity or irregular shape [27,28].

The average number of ultrasonographic features was significantly higher in TCs with unfavorable histopathological characteristics (3.05–3.12) compared to those without (2.70–2.88; *p* < 0.05). A cutoff of ≥3 suspicious features increased the likelihood of adverse pathology. Several studies have shown that the cumulative number strongly correlated with the likelihood of malignancy. It has been reported that ≥2 features markedly increase risk (specifically 89–97%), while another study reported malignancy rates of 48% with ≥3 features, compared to 5% with a single feature. EU-TIRADS category 5 also defines high-risk nodules in this regard, with malignancy risk rising as more of the suspicious patterns are present [36,37,38,46].

## 5. Conclusions

Our findings corroborate the literature regarding ultrasonographic features in thyroid imaging. An irregular appearance, alongside features such as hypoechogenicity, microcalcifications, and high vascularity, correlates with more unfavorable histopathological outcomes. Three positive ultrasonographic features correlate with a higher likelihood of unfavorable histopathology.

## Figures and Tables

**Table 1:** Study group (n = 724) demographics—clinical and pathological characteristics.

Feature		Quantity (Percentage)
Sex	M:F	110 (15%): 614 (85%)
Age	<55 years	404 (56%)
	≥55 years	320 (44%)
Type of thyroidectomy	Total	516 (71%)
	Hemi (Lobectomy)	208 (29%)
Reoperation procedure	Yes	195 (27%)
	No	529 (73%)
FNAB Result	Malignant or suspicious (Bethesda V/VI)	705 (97%)
	Indeterminate (Bethesda III/IV)	15 (2%)
	Benign (Bethesda II)	4 (1%)
Histological type of TC	Papillary TC	629 (87%)
	Follicular TC	36 (5%)
	Medullary TC	26 (4%)
	Anaplastic TC	17 (2%)
	Lymphoma	5 (<1%)
	Secondary lesion	4 (<1%)
	Squamous cell tumor	3 (<1%)
	Sarcoma	3 (<1%)
	Myeloma	1 (<1%)
TNM stage	I	539 (74%)
	II	112 (16%)
	III	36 (5%)
	IV	37 (5%)
pT stage	pT1a	292 (40%)
	pT1b	287 (40%)
	pT2	85 (12%)
	pT3	26 (3%)
	pT4a	12 (2%)
	pT4b	22 (3%)
pN stage	pN0	485 (67%)
	pN1a	184 (25%)
	pN1b	19 (3%)
	pNx	36 (5%)
pM stage	pM0	582 (80%)
	pM1	39 (5%)
	pMx	103 (15%)

**Table 2:** Chi-square test analyses of the association between ultrasonographic and histopathological TC features. A *p*-value of <0.05 represents a statistically significant difference.

	Extrathyroidal Extension	Capsular Invasion	Vascular Invasion	Lymph Node Metastases
**Hypoechogenicity**	*p* < 0.05	*p* < 0.05	*p* < 0.05	*p* < 0.05
**Microcalcifications**	*p* < 0.05	*p* < 0.05	*p* < 0.05	*p* < 0.05
**High Vascularity**	*p* < 0.05	*p* < 0.05	*p* < 0.05	*p* < 0.05
**Irregular Tumor Shape**	*p* < 0.05	*p* < 0.05	*p* < 0.05	*p* < 0.05

**Table 3:** Mann–Whitney U test analyses of average number of ultrasonographic TC features. A *p*-value of <0.05 represents a statistically significant difference.

Histopathological Feature	Average Number of Ultrasonographic Features (Positive Result of Histopathology)	Average Number of Ultrasonographic Features (Negative Result of Histopathology)	*p*-Value
Extrathyroidal Extension	3.12	2.78	<0.05
Capsular Invasion	3.05	2.72	<0.05
Vascular Invasion	3.06	2.70	<0.05
Lymph Node Metastases	3.08	2.88	<0.05

## Data Availability

The datasets used and/or analyzed during this research are available from the corresponding author upon reasonable request.
